# RETRACTED: Ahmed et al. Application of Nanopharmaceutics for Flibanserin Brain Delivery Augmentation Via the Nasal Route. *Nanomaterials* 2020, *10*, 1270

**DOI:** 10.3390/nano14020174

**Published:** 2024-01-12

**Authors:** Osama A. A. Ahmed, Usama A. Fahmy, Shaimaa M. Badr-Eldin, Hibah M. Aldawsari, Zuhier A. Awan, Hani Z. Asfour, Ahmed K. Kammoun, Giuseppe Caruso, Filippo Caraci, Anas Alfarsi, Raniyah A. Al-Ghamdi, Rawan A. Al-Ghamdi, Nabil A. Alhakamy

**Affiliations:** 1 Department of Pharmaceutics, Faculty of Pharmacy, King Abdulaziz University, Jeddah 21589, Saudi Arabia; oaahmed@kau.edu.sa (O.A.A.A.); smbali@kau.edu.sa (S.M.B.-E.); haldosari@kau.edu.sa (H.M.A.); mr_alfarsi8@hotmail.com (A.A.); nalhakamy@kau.edu.sa (N.A.A.); 2 Advanced Drug Delivery Research Group, Faculty of Pharmacy, King Abdulaziz University, Jeddah 21589, Saudi Arabia; 3 Department of Pharmaceutics and Industrial Pharmacy, Faculty of Pharmacy, Cairo University, Cairo 11562, Egypt; 4 Department of Clinical Biochemistry, Faculty of Medicine, King Abdulaziz University, Jeddah 21589, Saudi Arabia; zawan@kau.edu.sa; 5 Department of Medical Microbiology and Parasitology, Faculty of Medicine, King Abdulaziz University, Jeddah 21589, Saudi Arabia; hasfour@kau.edu.sa; 6 Department of Pharmaceutical Chemistry, Faculty of Pharmacy, King Abdulaziz University, Jeddah 21589, Saudi Arabia; akammoun@kau.edu.sa; 7 Oasi Research Institute-IRCCS, Via Conte Ruggero, 73, 94018 Troina (EN), Italy; gcaruso@oasi.en.it (G.C.); carafil@hotmail.com (F.C.); 8 Department of Drug Sciences, University of Catania, 95125 Catania, Italy; 9 Ibn Sina National College for Medical Studies, Clinical Pharmacy Department, Jeddah 22421, Saudi Arabia; ranoabg@hotmail.com; 10 Ibn Sina National College for Medical Studies, Jeddah 22421, Saudi Arabia; rawanaasg3@gmail.com; 11 Center of Excellence for Drug Research and Pharmaceutical Industries, King Abdulaziz University, Jeddah 21589, Saudi Arabia

The Journal retracts the article “Application of Nanopharmaceutics for Flibanserin Brain Delivery Augmentation Via the Nasal Route” [1].

Following the publication, concerns were brought to the attention of the Editorial Office regarding the overlapping figures between publications [1,2], representing different experimental conditions. More specifically, subfigures B and D of Figure 6 in publication [2] are duplicated as subfigures D and C of Figure 6 in publication [1], respectively.

The authors cooperated with the investigation and commented that the mistake occurred while finalizing the figures for both studies, and the wrong image was selected for publication [1]. The authors also provided raw data and other documents related to the histopathological experiments in the reported study. However, the Editorial Board and Editor in Chief determined that the evidence and explanation provided were insufficient to justify the overlap observed, and inconsistencies in the raw data provided meant that serious doubts remained regarding the reliability of the data provided. As a result, the Editorial Board and Editor in Chief were unable to confirm the reliability of the findings and subsequently decided to retract this paper. 

This retraction was approved by the Editor in Chief of the journal *Nanomaterials.*

The authors did not agree to this retraction.
